# Extramammary Paget’s Disease in the Genital Area of a Male: A Case Report and Review of the Literature

**DOI:** 10.3389/fonc.2021.713786

**Published:** 2021-11-04

**Authors:** Dan Zhao, Bo-ping Wen, Sen-yin Xu

**Affiliations:** ^1^ Department of Medical Ultrasound, Hangzhou Red Cross Hospital, Zhejiang Hospital of Integrated Traditional Chinese and Western Medicine, Hangzhou, China; ^2^ Deparment of Ultrasound, Zhejiang Hospital, Hangzhou, China

**Keywords:** extramammary Paget disease (EMPD), conventional ultrasonography (US), contrast-enhanced ultrasound (CEUS), scrotum, imaging findings

## Abstract

Extramammary Paget’s disease (EMPD) is an uncommon intraepithelial malignancy that is rarely found in the male. Currently, there is very little knowledge pertaining to EMPD imaging, particularly in cases that involve the scrotum. Here, a 67-year-old man with lichenification on his left scrotum confirmed to be EMPD was reviewed. Bloodwork did not return a positive result, but syphilis-specific antibodies were found. Conventional high-frequency ultrasound (US) and contrast-enhanced ultrasound (CEUS) imaging were utilized to determine the lesion size and blood perfusion. In the present case, the lesion’s size and involvement were vividly depicted by CEUS, while results obtained by conventional US were grossly underestimated. Consequently, multimodal imaging assessment is likely to provide more accurate diagnoses for uncommon diseases, such as EMPD, and to aid in clinical decision-making.

## Introduction

Paget disease was named after Sir James Paget, who first described an eczema-like lesion around the nipple associated with underlying breast malignancy in 1874 (1–3). Extramammary Paget disease (EMPD) is a rare intraepithelial malignancy that originates from the skin or the regions rich in apocrine glands. It is rare in males, although the disease affects both males and females (4, 5). The most common location of EMPD is the vulvar, followed by the perianal region, and finally male genitalia (6, 7). Moreover, Crocker first described EMPD in the penis and scrotum in 1889 (8, 9).

The EMPD typically appears as a non-specific erythematous, pruritic, and scaly eroded plaque, with lichenification and ulcerations (10, 11). It is often misdiagnosed as common cutaneous diseases, such as eczema, psoriasis, dermatitis, and lichen sclerosis (10, 12, 13), with delayed appropriate treatment as a result.

High-frequency ultrasonography (US) is an affordable, non-invasive, radiation-free, and effective diagnostic procedure that is often used for imaging-based diagnosis and preoperative assessment of soft tissue lesions (14). However, to our knowledge, there is little to no prior research pertaining to the contrast-enhanced ultrasound (CEUS) of EMPD. Herein, we report the US and CEUS findings of an EMPD lesion on the scrotal wall.

## Case Presentation

**Figure 1:**
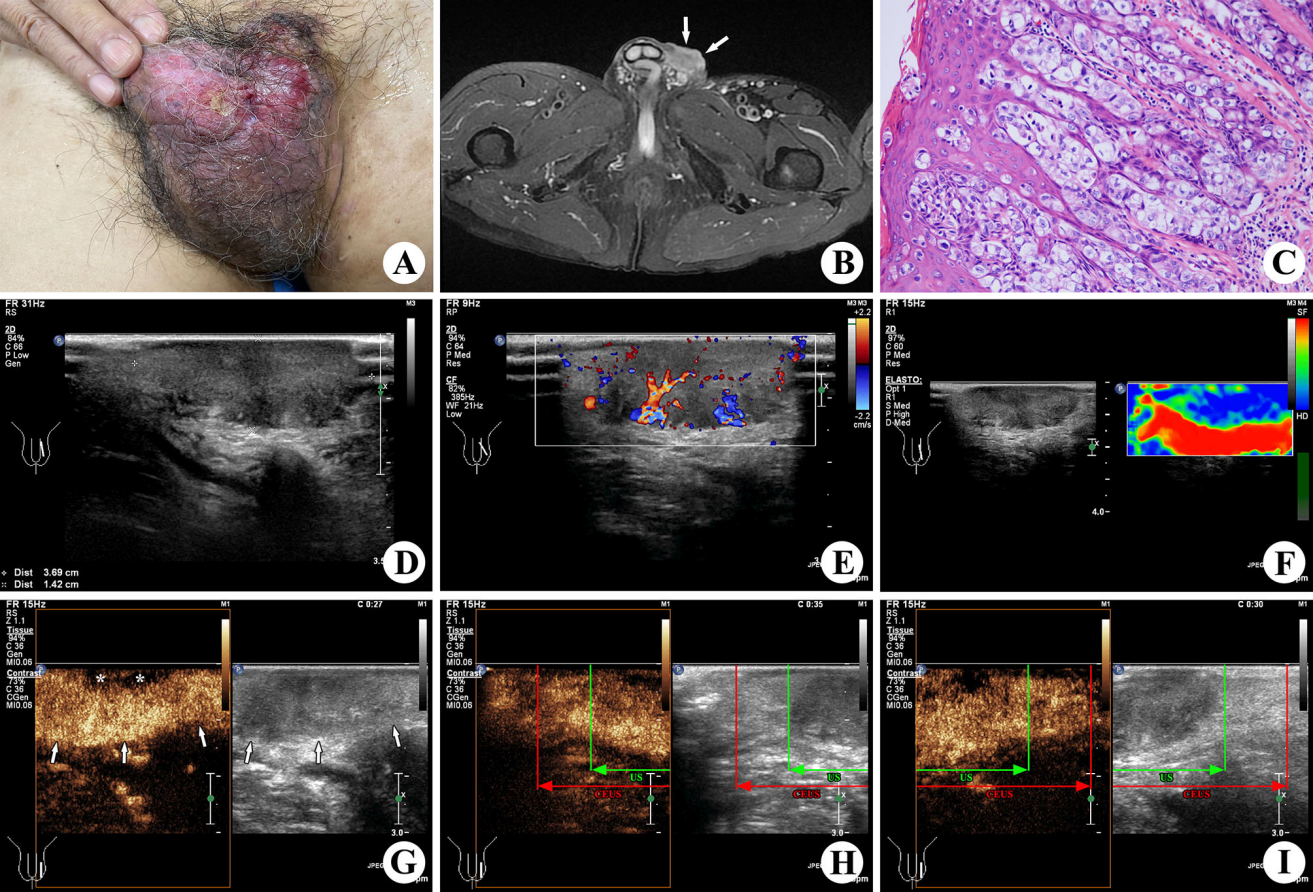
A 67-year-old patient with EMPD. An ulcerative and eczematous lesion was shown on his left scrotum **(A)**. A lesion with irregular margin (↑) was found at his left scrotum in T2WI fat-suppressed sequences of MR **(B)**. Scattered epidermal Paget cells with a pleomorphic and hyperchromatic nucleus and abundant clear cytoplasm were located throughout the epidermis in nest-like structures in histopathologic examination of the excised specimen (×200; H&E stain) **(C)**. In the preoperative conventional US, a 3.6 × 2.9 × 1.4 cm lesion on the left side scrotal wall appeared to be hypoechogenicity, infiltrative margin, and presence of profuse vascularity **(D, E)**. Elastography showed blue color in and around the lesion, which was valued to be hard in a score of 4 **(F)**. The preoperative CEUS showed hyper-enhancement (↑) in the main part and non-enhancement (*) in the superficial area of the lesion **(G)**, which was larger than that shown in the conventional US. In the upper part of the lesion **(H)**, the green line represents the upper boundary confirmed by the conventional US, and the red line represents the upper boundary confirmed by CEUS. In the lower part of the lesion **(I)**, the green line represents the lower boundary confirmed by the conventional US, and the red line represents the lower boundary confirmed by CEUS.

A lesion was found on the left scrotal wall of a 67-year-old man 11 months ago, followed by ulceration at the surface of the lesion 6 months later. The patient was in good health before and had no history of hepatitis, diabetes, tuberculosis, or cancer. He had no history of medical, family, psychosocial, or genetic problem neither. Hard and diffuse eczematous changes and ulcerations were revealed on his left scrotum by physical examination (**Figure 1**). Palpations of his testis and epididymis were normal, along with no swelling or palpable mass in his bilateral groins. Nothing notable was found from bloodwork and conventional chest CT. The bloodwork did however show syphilis-specific antibodies. According to the physical examination and patient history, it was initially diagnosed with a common skin disease, such as dermatitis or eczema. Since no response to a diagnostic therapy of topical ointment was found thereafter, further examinations were subsequently ordered. With relevant guidelines and regulations complied, the work was approved by the Ethical Committee of Zhejiang Hospital of Integrated Traditional Chinese and Western Medicine and informed consents were obtained from the patient.

The conventional high-frequency US examination of the lesion was performed, using the Philips iU22 Ultrasound machine (Philips Healthcare, Bothell, WA, USA) with the L12-5 linear array transducer (frequency range, 5.0–12.0 MHz). An isoechoic to hypoechoic lesion measuring 3.6 × 2.9 × 1.4 cm was found on his left-sided scrotal wall, with an infiltrative margin and cutaneous invasion (including epidermis, dermis, and hypodermis invasion). Color Doppler ultrasound showed a profuse signal of intralesional vascularity, and the resistance index was 0.76. Elastography was performed to assess the lesion stiffness, which was valued to be hard in a score of 4 according to the Itoh score system (15). No abnormality was found in the testis, epididymis, or bilateral groins.

A lesion with hypointensity on T1WI and heterogeneous hyperintensity on T2WI was found at his left scrotal wall by magnetic resonance (MR) (1.5 T, SIGNA Explorer, GE, Tianjin, China). However, the extent of lesion invasion was not accurately measured in such uneven location.

Subsequently, CEUS was performed with the L9-3 linear array transducer (frequency range, 3.0–9.0 MHz) and low acoustic power (mechanical index, 0.06) for further confirming the extent of the lesion before surgery. The examination was carried out by an experienced radiologist after intravenous bolus injection of 4.8 ml SonoVue™ (Bracco Diagnostics Inc., Italy), flushed with 10 ml of a physiological saline solution. Rapid enhancement of the lesion was shown in CEUS, which indicated a malignant tumor. The lesion measuring 5.6 × 4.9 × 1.4 cm with hyper-enhancement in the main area and non-enhancement in the superficial area was shown in all stages, which was larger than that shown on conventional US. Then, we made marks on the skin before surgical resection.

Based on all clinical data and imaging findings, it was highly suspected as a skin malignant tumor before surgery, such as squamous cell carcinoma or adenocarcinoma of skin. Dermatosis associated with syphilis was also a possibility. Finally, a histopathological result of EMPD was confirmed after conducting biopsy on the excised lesion. It found scattered epidermal Paget cells with a pleomorphic and hyperchromatic nucleus and abundant clear cytoplasm, which were located throughout the epidermis in nest-like structures, involving full skin layers and subcutaneous soft tissue (including nerves and blood vessels). The present case showed all morphologic features of EMPD including positive immunohistochemical staining for CEA, CK7, and GCDFP-15. Moreover, the volume of the lesion on the gross specimen was 5.5 × 5.0 × 1.3 cm, which matched the result of CEUS.

The patient did not receive radiotherapy or chemotherapy after surgery and has been closely followed up to now.

## Discussion

Diagnosis and treatment of EMPD are often delayed after the patient’s initial presentation of the disease, which is likely related to the diversity of the symptoms and the rarity of the disease, leading to poor prognosis of the disease such as expansion, deep invasion, and distant metastases (16). A median time delay before the correct diagnosis of EMPD was found to be 2 years (8). The clinical differential diagnosis for EMPD is very broad, because the underlying squamous cell carcinoma or adenocarcinoma of skin usually mimics it (11, 17). In the present case, the positive result of the syphilis-specific antibody also confused the diagnosis. For patients that present with non-specific, ulcerative, and eczematous lesions in scrotal skin and cannot be cured by conventional treatment, EMPD diagnosis should be considered. Moreover, a surgical resection and biopsy of the lesion should be performed as early as possible (2, 3, 18). The accurate assessment of the involving extent before operation may be the key factor for the success of surgical resection.

Ultrasonography aids EMPD diagnosis and preoperative assessment by giving the anatomic information, illustrating internal blood flow, and giving a lymph node assessment of metastasis (19). With the development of technology, high-resolution US provides a preferred method to characterize and assess the extent of cutaneous lesions (14). In this case, the lesion was inaccurate in measuring the extent of invasion by MR due to the uneven site of the disease. US elastography is a promising and effective imaging-based protocol for tissue stiffness assessment, which can potentially show the nature of malignancy (20). In the lymph node, liver, kidney, and other organs, the nature of the lesion was accurately assessed through the characterization of blood perfusion by CEUS imaging (21–24). In the present case, the microvascular perfusion of the lesion and the extent of the invasion were clearly shown by CEUS. The non-enhancement in the superficial area of the lesion indicated the ulcerative area of the lesion. It was critical that the actual lesion involvement of the gross specimen was distinctly shown by the CEUS, which was obviously underestimated by conventional US. The enhanced pattern of EMPD might be similar to breast cancer, in which the area of enhancement was larger than that shown on the conventional US thanks to the tumor invasion resulting from angiogenic factors (25). To our best knowledge, the present case of EMPD is the earliest report on CEUS findings, which concluded that multimodal imaging assessment can provide an accurate diagnosis for uncommon diseases, like EMPD, to help in clinical decision-making and preoperative lesion assessment.

## Conclusions

Conventional high-frequency US and contrast-enhanced ultrasound CEUS were performed to assess the lesion size and blood perfusion. By utilizing CEUS, the actual lesion involvement according to the gross specimen was shown to be more significant than conventional non-enhanced US.

## Data Availability Statement

The raw data supporting the conclusions of this article will be made available by the authors, without undue reservation.

## Ethics Statement

The studies involving human participants were reviewed and approved by the institutional research ethics committee of Hangzhou Red Cross Hospital. The patients/participants provided their written informed consent to participate in this study. Written informed consent was obtained from the individual(s) for the publication of any potentially identifiable images or data included in this article.

## Author Contributions

DZ, B-pW, designed, organized, and supervised the study. DZ, B-pW, drafted the manuscript. DZ, and S-yX analyzed the literature. S-yX revised the manuscript. DZ, B-pW, and S-yX participated in the revision. All authors contributed to the article and approved the submitted version.

## Funding

This work was supported by grants from the Basic Public Welfare Research Program of Zhejiang Province (No. LGF20H180003) and Zhejiang Medical and Health Science and Technology Project (No. 2019KY511).

## Conflict of Interest

The authors declare that the research was conducted in the absence of any commercial or financial relationships that could be construed as a potential conflict of interest.

## Publisher’s Note

All claims expressed in this article are solely those of the authors and do not necessarily represent those of their affiliated organizations, or those of the publisher, the editors and the reviewers. Any product that may be evaluated in this article, or claim that may be made by its manufacturer, is not guaranteed or endorsed by the publisher.
